# Cryopreservation and Flow Cytometric Analysis of Ovarian Tissue in Murray River Rainbowfish, *Melanotaenia fluviatilis*

**DOI:** 10.3390/ani12060794

**Published:** 2022-03-21

**Authors:** Nicola Rivers, Jonathan Daly, Robert Jones, Peter D. Currie, Peter Temple-Smith

**Affiliations:** 1 Department of Obstetrics and Gynaecology, School of Clinical Sciences, Monash University, Clayton, VIC 3168, Australia; peter.temple-smith@monash.edu; 2 The Australian Frozen Zoo, Clayton, VIC 3168, Australia; jondaly@zoo.nsw.gov.au; 3 Taronga Conservation Society Australia, Mosman, NSW 2088, Australia; 4 The Aquarium Vet, Moorabbin, VIC 3189, Australia; rob@theaquariumvet.com; 5 Australian Regenerative Medicine Institute, Clayton, VIC 3800, Australia; peter.currie@monash.edu

**Keywords:** fish, biobanking, cryopreservation, ovary, conservation

## Abstract

**Simple Summary:**

Freshwater fish populations are in global decline, with many Australian freshwater species expected to become extinct in the next twenty years. The storage of reproductive cells and tissues at extremely cold temperatures in bio-banks known as “Frozen Zoos”, allows for the indefinite storage of genetic material, meaning that in the event of an extinction, we have a genetic blueprint available to produce new individuals and reintroduce a species into the wild. Here we have developed a cryopreservation protocol for the storage of ovarian tissue from the threatened Murray River Rainbowfish. Many Australian freshwater fish species are threatened with extinction, our methodology provides a framework for the conservation of other fish species in Australia and globally.

**Abstract:**

Freshwater fish populations are declining with many small, Australian fish species at risk of extinction within the next twenty-years. Cryopreservation of reproductive cells and tissues makes it possible to reproduce individuals from a species even after they are extinct in the wild. We describe the successful cryopreservation of ovarian tissue in the Murray River Rainbowfish, *Melanotaenia fluviatilis* (Order: Atheriniformes). Histology showed that oogonia are 13.70 µm ± 1.75 µm in size, stain positive for germ-line marker Vasa, and represent approximately 2.29 ± 0.81% of cells in the ovary. Flow cytometry was used to analyse ovarian cell suspensions, requiring an optimised tissue digestion protocol. We found that 0.25% trypsin with 1.13 mM EDTA produced cell suspensions with the highest viability (76.28 ± 4.64%) and the highest number of cells recovered per gram of tissue (1.2 × 10^8^ ± 4.4 × 10^7^ cells/g). Subsequent sorting of ovarian cell suspensions by flow cytometry increased oogonial cells in suspension from 2.53 ± 1.31% in an unsorted sample to 5.85 ± 4.01% in a sorted sample (*p* = 0.0346). Cryopreservation of ovarian tissue showed DMSO-treated samples had higher cell viability post-thaw (63.5 ± 18.2%) which was comparable to fresh samples (82.5 ± 7.1%; *p* = 0.36). Tissue cryopreserved in 2.0 M DMSO had the highest cell viability overall (76.07 ± 3.89%). This protocol could be applied to bio-banking programs for other species in the Melanotaeniidae, and perhaps species in other families and orders of Australian fish.

## 1. Introduction

Australian freshwater fish species are under threat of extinction due to continued habitat degradation and predation by invasive species [1]. Small fish species, in particular, have been reported to be at a high risk of extinction within the next twenty-years without swift intervention to protect their natural habitat [2]. However, such interventions can take time to be effective during which species remain vulnerable to further declines. The use of bio-banking is an ex situ means of securing fish genetics in the event of a sudden drop in genetic diversity or species extinction [3,4]. The cryopreservation of reproductive cells and tissues provides scientists an opportunity to reproduce and reintroduce individuals from a species even after they have gone extinct in the wild. While cryopreservation has been successful in preserving cells from male fish including sperm [5] and spermatogonial cells [6], there has been very limited success in the cryopreservation of female reproductive cells and tissues, with no reports in Australian fish species. 

Fish oocytes are large and contain a high percentage of lipid and water which results in poor cryoprotectant permeability and a high risk of cryo-injury during cryopreservation [7]. For these reasons, the recovery of viable, fertilisable oocytes post-thaw has been challenging. Fish embryos face similar limitations [8] and even with technological advancements including the injection of cryoprotectants into the yolk [9] and the use of gold nanoparticles and laser warming to improve thawing speed [10,11], post-thaw viability in fish embryos remains low. Cryopreservation has been somewhat more successful in smaller, immature oocytes [12], however production of mature, fertilisable oocytes from immature, cryopreserved oocytes has not yet been achieved in vitro [13]. In the absence of reliable oocyte and embryo cryopreservation methods, there are only limited opportunities to secure maternal fish genomes in biobanks. Without a robust method for the successful cryopreservation and warming of fish oocytes, the applications of sperm cryopreservation are limited to species in which mature oocytes are able to be collected from living fish for fertilisation [5]. In the context of endangered species, this poses a significant limitation as the purpose of bio-banking is often to resurrect populations of significantly reduced or extinct species, at which point accessing spawning females may be extremely difficult or even impossible. The cryopreservation of ovarian tissue containing oogonia, the early cell stage of oogenesis, has become an increasingly popular target for the bio-banking of genetics of female fish. Cryopreservation of ovarian tissue has been reported in several species, including the common carp (*Cyprinus carpio*) [14], Piracanjunba (*Brycon orbignyanus*) [15], sea trout (*Salmo trutta*) [16], American paddlefish (*Polyodon spathula*) [17], Siberian sturgeon (*Acipenser baerii*) [18] and the endangered cyprinid honmoroko (*Gnathopogon caerulescens*) [19], representing families within the orders of Cypriniformes, Characiformes, Salmoniformes and Acipenseriformes. 

Oogonia are relatively small, simple cells that are capable of differentiating into mature oocytes via oogenesis. They have the advantage of being able to be isolated from the ovaries of fish and cryopreserved at any life stage, regardless of their sexual maturity or spawning period. While oogonia are undifferentiated germ cells, they can be differentiated into viable oocytes via a surrogate species using a method commonly referred to as germ cell transplantation (GCT) or cell surrogacy [20]. Briefly, gonial cells extracted from cryopreserved gonadal tissue of a threatened species, are injected into a sterile surrogate where they incorporate into the gonad, re-establish gametogenesis and eventually produce donor derived gametes. 

Australia is home to a number of threatened freshwater fish species. Recent fish kills in the country’s largest freshwater river system, the Murray Darling Basin [21], highlight the need for ex situ conservation methods including biobanking. To our knowledge there has been no investigation into the potential use of ovarian tissue cryopreservation in Australian fish species. In collaboration with the Australian Frozen Zoo, a Melbourne based biobanking initiative that has been operating for the past twenty-five years, we have been investigating the potential application of gonad cryopreservation in Australian fish species. We previously described successful cryopreservation of testicular tissue in the Murray River Rainbowfish (*Melanotaenia fluviatilis*) [22] from the family Melanotaeniidae (order Atheriniformes), a group of small freshwater fish species endemic to areas across Australia and surrounding regions including New Guinea. While *M. fluviatilis* is listed as threatened within the state of Victoria [23], this species also has several critically endangered and endangered relatives including the Running River Rainbowfish (*M. splendida. nov*), the Slender Rainbowfish (*M. gracilis*), the Lake Eacham Rainbowfish (*M. eachamensis*), and the Utchee Creek Rainbowfish (*M. utcheensis*) [24]. 

Here we describe the successful cryopreservation of ovarian tissue in *M. fluviatilis*; a protocol (data set in Supplementary Materials) that could be applied to bio-banking programs for other species in Melanotaeniidae, and perhaps species in other families and orders of Australian fish. We have also investigated the use of flow cytometry as a method for isolating and analysing specific ovarian cell types in ovarian tissue suspensions. In the absence of successful oocyte or embryo cryopreservation protocols, the cryopreservation and storage of ovarian tissue from fish species presents an accessible target for current biobanking programs. 

## 2. Materials and Methods

### 2.1. Animal Handling and Ethics Approval

Female *M. fluviatilis* obtained from an aquarium supplier, Aquarium Industries (Epping, VIC, Australia), were held at 25 °C ± 1 °C on a 12:12 light-dark cycle. At the time of collection, fish weighing 1.33 g ± 0.72 g and 4.27 cm ± 0.7 cm in length (standard length) were killed by anaesthetic overdose by submersion in 175 mg/L of AQUI-S (Primo Aquaculture, Narangba, QLD, Australia) diluted in system water for 20 min. A total of thirty-eight (*n* = 38) female *M. fluviatilis* were used, with specific sample sizes for each experiment included below. Based on their length, the age of fish used in this study is estimated to be between one to two years old [25]. Death was confirmed by manual destruction of the brain. Gonads weighing 0.047 g ± 0.04 g were removed and placed into chilled phosphate–buffered saline (PBS; pH 7.8). All animal handling and experimental procedures were performed in accordance with animal ethics application MMCB/2017/39 approved by the Animal Ethics Committee B at Monash Medical Centre and conducted in accordance with the Australian Code of Practice for the Care and Use of Animals for Scientific Purposes.

### 2.2. Histology & Immunohistochemistry

Whole ovaries from five (*n* = 5) *M. fluviatilis* were fixed in 10% neutral buffered formalin (Merck, VIC, Australia) for 48 h and processed by the Monash Histology Platform, which included standard haematoxylin and eosin staining. Unstained sections were stained for Vasa, to identify oogonia, using a zebrafish (*Danio rerio*) specific anti-Vasa antibody (Sapphire Bioscience Pty. Ltd., Redfern, NSW, Australia) and counter stained with Hoechst (ThermoFisher Scientific, Waltham, MA, USA) as described in Rivers et al., 2020a. Briefly, de-paraffinised sections were rehydrated before antigen retrieval in 10 mM citrate buffer (pH 6). Sections were blocked with CAS Block (Invitrogen, Waltham, MA, USA) for one hour followed by incubation with anti-Vasa antibody (1:200) in diluent, 5% bovine serum albumin (BSA) in PBS, at 4 °C overnight. Sections were incubated with secondary antibody, Alexa Fluor 488-conjugated goat anti-rabbit IgG (1:500), and Hoechst nuclear counterstain (1:1000) in diluent before cover-slipping for imaging. Images were captured using the EVOS FL Auto 2 Imaging system (ThermoFisher Scientific) and Olympus BX43 Upright Microscope with an X-Cite Series 120 Q laser (Lumen Dynamics, Mississauga, ON, Canada). Sizes of oogonia were measured using cellSens Standard imaging software (Software version: 1.16, build 15404, Olympus, Tokyo, Japan) and images were analysed in FIJI [26]. An estimation of the proportion of oogonia present in the ovary in comparison to total ovarian cell number was determined by counting the number of oogonia, based on size and Vasa-positive staining, and the total number of cells across five randomly sampled fields taken at 20× magnification with at least 250 cells present per field. 

### 2.3. Cryopreservation and Thawing

In experiment 1, the efficacy of two different permeating cryoprotectants was tested. Whole gonads from twelve (*n* = 12) female *M. fluviatilis* were divided across four treatment groups: fresh control, dimethyl sulfoxide (DMSO) treated, ethylene glycol (EG) treated, or negative control with each treatment assessed in triplicate. Ovaries in the fresh control group, were transferred into separate 1.2 mL CryoTubes with 1 mL trypsin-based digestion media prior to flow cytometry as described in Section 2.4 and Section 2.5. Ovaries undergoing cryopreservation were placed in a cryotube with 1ml of cryomedia containing a permeating cryoprotectant, either DMSO or EG, at a concentration of 1.3 M with 0.1 M trehalose (Merck, Darmstadt, Germany) and 1.5% BSA (Bovogen Biologicals Pty. Ltd., Keilor East, VIC, Australia) in a mixed salt extender previously described by [27] (~300 mOsm, pH 7.8). Negative control samples contained all cryomedia components excluding the permeating cryoprotectant. 

In experiment 2, DMSO was tested at a concentration of 1.0 M, 1.6 M and 2.0 M with each treatment assessed in triplicate (*n* = 9), with the same base media components as in experiment 1. Both experiments followed the same cooling and thawing method in which samples were equilibrated on ice for one hour and then cooled them at a rate of −1 °C/minute in a CoolCell LX cell freezing container (Corning, Glendale, AZ, USA) in a −80 °C freezer for at least 3 h before plunging them into liquid nitrogen where they were stored for at least 24 h before thawing.

Cryopreserved tissue was thawed in a 30 °C water bath for two minutes before rehydration in three changes of handling media (Eagles minimum essential media (EMEM, SigmaAldrich, St. Louis, MO, USA) supplemented with 5% FBS, ThermoFisher Scientific, Waltham, MA, USA; and 25 mM HEPES, ThermoFisher Scientific; pH 7.8) for 20 min per change.

### 2.4. Preparation of a Single Cell Suspension

A single cell suspension was produced from fresh and frozen samples prior to flow cytometry. To optimize the cell suspension protocol, the ovary of five (*n* = 5) *M. fluviatilis* were split into three portions and assigned to one of the following cell suspension protocols—(i) mechanical crushing with a glass tissue grinder, (ii) digestion with 0.2% collagenase in PBS at 29 °C ± 1 °C for 2 h, or (iii) digestion in 0.25% trypsin with 1.13 mM EDTA in PBS for 30 min on ice and 1 h at room temperature. Digestion reactions using trypsin were stopped by addition of 2% serum. All samples were then filtered through 40 µm nylon mesh and cells were pelleted by centrifugation in a microfuge at 7000 rpm for 1 min at room temperature and resuspended in 1.13 mM EDTA in PBS for analysis by flow cytometry. The viability of cells 10 µm and larger was determined using the flow cytometer, and the total number of cells recovered per gram of tissue was determined using a haemocytometer. 

### 2.5. Flow Cytometry

A set of size-specific beads (16.5 µm, 10.2 µm, 7.56 µm, 5.11 µm, 3.3 µm, Spherotech, Lake Forest, IL, USA) were run on the FACS Aria Fusion flow cytometer (BD Biosciences, Macquarie Park, NSW, Australia) prior to sample analysis to provide an approximate scale to determine the size of cells in suspension. Oogonia are the primary target when cryopreserving ovarian tissue. As such, it was important to determine the number of these cells able to be isolated from the single cell suspension using flow cytometry. To do this, ovaries from five (*n* = 5) *M. fluviatilis* were pooled into a single cell suspension and split into ten 500 uL samples, a 100 µL aliquot was taken to analyse the unsorted cells and the remaining sample was run through a flow cytometer. A gate was set to sort events approximately 10 µm and higher, capturing the approximate range of oogonia sizes as determined by histology. Live cells in the sorted and unsorted samples were imaged and measured to determine the range of sizes captured by the gate. The cells were then fixed in 2% formalin and dried onto SuperFrost plus slides overnight at 37 °C. Slides were stained with anti-Vasa antibody and Hoechst and imaged. Ten fields were selected randomly per sample and the number of Vasa-positive and Vasa-negative cells were counted to determine the average proportion of Vasa positive, oogonia in each sample analysed.

### 2.6. Viability Analysis

Viability analysis was performed for all samples from Section 2.3 and Section 2.4. At time of analysis, single cell suspensions were split across four 5 mL sample tubes in preparation for flow cytometry. Viability was determined using two vitality stains, Propidium Iodide (PI), a membrane impermeable dye that tags non-viable cells with membrane damage, and a membrane permeable counter-stain SYBR-14. One sample was kept as an unstained control, and single dye controls were prepared for SYBR14 and PI, with the PI control sample flash frozen in liquid nitrogen three times to ensure a high number of dead cells for analysis. Samples were incubated with SYBR14 (5 µL/mL) for 5 min in the dark, followed by a second 5 min incubation with PI (5 µL/mL). The flow cytometer was set to capture at least 50,000 events for each sample and flow cytometry output was analysed in FlowJo^TM^ Software for Mac [28]. The viability of each sample was determined by the proportion of SYBR14 positive only events within all events captured by the sorting gate. 

### 2.7. Statistical Analysis

Data analysis was performed in Prism 8 for MacOS (version 8.4.2) with a *p*-value of 0.05 considered statistically significant; all data are reported as mean ± standard deviation unless otherwise specified. Cell suspension viability, suspension cell recovery and cryopreservation viability data were all found to meet the assumptions of normality and variance using the Shapiro-Wilk test and the Brown-Forsythe test, respectively; data were then analysed by one-way ANOVA with Tukeys post hoc test. Comparison of the proportion of Vasa-positive cells in unsorted and sorted ovarian cell suspensions met the assumption of normality using the Shapiro-Wilk test and were analysed using an un-paired, two-tailed *t*-test. 

## 3. Results

### 3.1. Histology of M. fluviatilis Ovary

**Figure 1:**
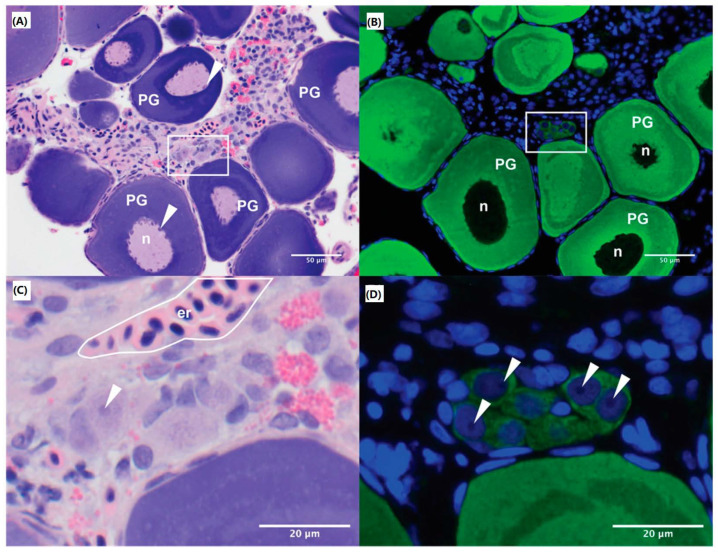
Histology of M. fluviatilis ovary. Ovarian tissue section stained with haemotoxylin and eosin (**A**) and anti-Vasa antibody (green fluorescence) and Hoechst nucleic counter stain (blue fluorescence) (**B**). A number of oocytes in the primary growth stage (PG) are present and a region of interest surrounded by a white box (**C**,**D**) shows a cluster of oogonia embedded within the germinal epithelium. Er: erythrocytes; n: nucleus; PG: primary growth stage oocyte; White arrow: nucleoli.

Oogonia are hypochromatic, light-staining cells found in clusters embedded in the germinal epithelium. They can be difficult to visualise using haematoxylin and eosin alone, but are readily differentiated from the surrounding somatic cells when stained with anti-Vasa antibody (Figure 1). Measurements of the different stages of oogenesis in the ovary showed oogonia to be approximately 13.70 µm ± 1.75 µm in size. Ovaries used for these experiments contained a range of oocyte maturation stages. Primary oocytes and early cortical alveolar stage oocytes were the most common germ cells in the ovary with oogonia representing approximately 2.29 ± 0.81% of the total number of ovarian cells. 

### 3.2. Producing a Single Cell Suspension and Identifying Germ Line Cells in Suspension

To analyse ovarian cells using flow cytometry, we first optimised a method to prepare single cell suspensions from fresh ovarian tissue by comparing cell recovery and viability from three isolation methods: a tissue crushing method and two enzyme digestion methods. Cell recovery and viability were assessed by the number of cells recovered per gram of tissue used to produce the suspension and the number of viable (live) cells in suspension over 10 µm in size on the flow cytometer.

**Figure 2:**
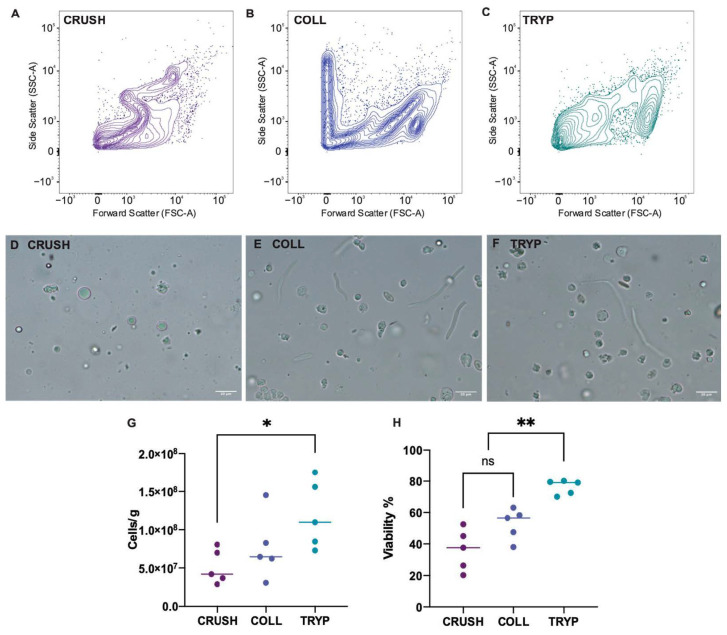
Validation of cell suspension protocol in M. fluviatilis ovarian tissue. The ovary from five (*n* = 5) M. fluviatilis were split into three segments and allocated to one of three treatments. Flow cytometry scatter plots for cell suspensions produced using the (**A**) crushing method, (**B**) collagenase digestion and (**C**) trypsin digestion. Images of live cell suspensions in (**D**) crushed, (**E**) collagenase and (**F**) trypsin treated samples. (**G**) The number of cells recovered/gram of tissue was significantly different between crushed and trypsin-treated samples (*p* = 0.0364, level of significance indicated by *), but not between crushed and collagenase-treated tissue (*p* = 0.5544) nor between collagenase-treated and trypsin-treated tissue (*p* = 0.2157; One-way ANOVA with Tukeys post hoc test). (**H**) The viability of cells captured in the 10 µm and larger region by flow cytometry was highest in trypsin-treated tissue (76.28 ± 4.64%; *p* < 0.007, level of significance indicated by **) but no difference was observed in cell viability between crushed and collagenase treated samples (*p* = 0.0557, non-significance indicated by “ns”; One-way ANOVA with Tukeys post hoc test).

Crushing ovarian tissue with a glass tissue grinder produced the lowest number of cells in suspension, 5.1 × 10^7^ ± 2.2 × 10^7^ cells/g, and a viability of 36.34 ± 13.19%. Digestion with collagenase resulted in a higher mean number of cells in suspension, 7.7 × 10^7^ ± 4.2 × 10^7^ cells/g, which was not statistically significant compared to crushed tissue, and a viability of 52.68 ± 9.96%. Digestion in 0.25% trypsin with 1.13 mM EDTA produced the best cell suspension out of the three treatments, with the highest viability at 76.28 ± 4.64% which was statistically significant to both crushed (*p* < 0.0001) and collagenase treated samples (*p* = 0.007). Trypsin treated tissue also resulted in the highest number of cells recovered per gram of tissue, 1.2 × 10^8^ ± 4.4 × 10^7^ cells/g, which was significantly different to crushed samples (*p* = 0.0364) but not significantly different to collagenase treated tissue (*p* = 0.2157). Flow cytometry plots also showed trypsin to have the most clearly resolved cell population (Figure 2).

**Figure 3:**
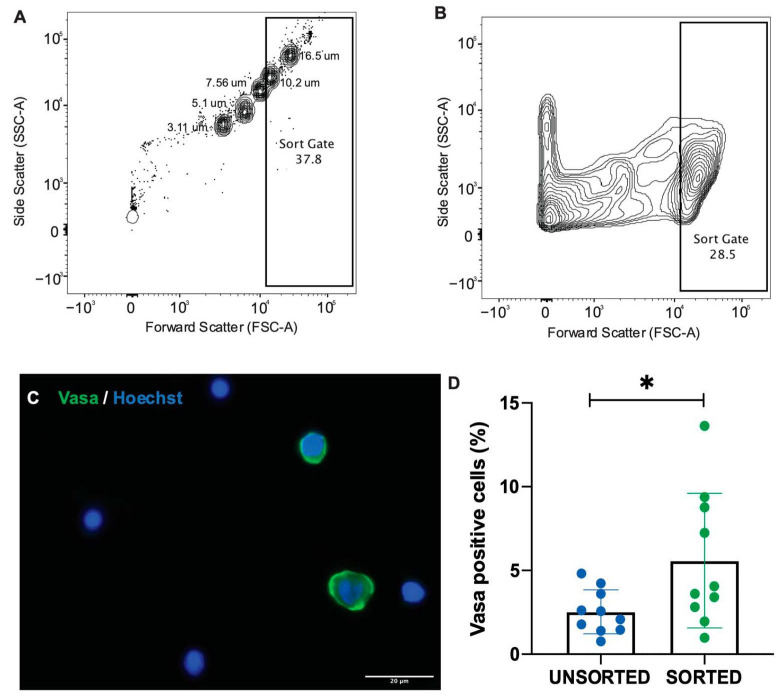
Flow cytometry-based cell sorting method. (**A**) A set of size-specific beads were used to give an approximation of particle size in relation to forward scatter (FSC), a gate was then set to capture events approximately 10 µm and larger. A pooled (*n* = 5) ovarian cell suspension was split across 10 samples and each sample was (**B**) sorted by a gate on the flow cytometer. (**C**) An example of Vasa-positive cells in a sorted cell sample. (**D**) The proportion of Vasa-positive cells after sorting was significantly higher compared to unsorted samples (5.85 ± 4.01% vs. 2.53 ± 1.31%, *p* = 0.0346, level of significance indicated by *; two-tailed *t*-test, data shown as mean ± standard deviation).

The purpose of cryopreserving ovarian tissue was to recover viable germ cells including oogonia capable of dividing and differentiating into viable oocytes via a surrogate. Our aim was to determine the most effective cell preparation method that captures the highest number of viable oogonia in comparison to ovarian cell suspension as a whole. We developed a flow cytometry-based sorting method using a set of size-specific beads to isolate a population of cells, 12 µm and larger, that we predicted would have a higher proportion of germ cells. Samples sorted by the flow cytometer were then stained and analysed for the proportion of Vasa-positive, oogonia in sorted and unsorted samples. Our gating method captured cell samples containing significantly higher concentrations of Vasa-positive, oogonia, than unsorted samples (5.85 ± 4.01% and 2.53 ± 1.31%, respectively; *p* = 0.0346; Figure 3).

### 3.3. Optimisation of Cryopreservation in M. fluviatilis Ovary

**Figure 4:**
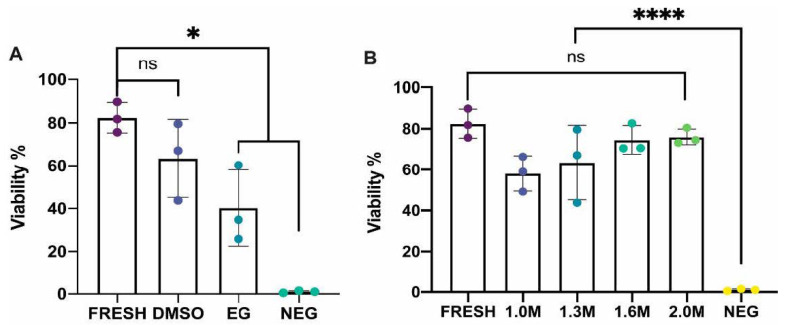
Comparison of ovarian cells viability between various concentrations of cryoprotectants and against fresh and negative controls. (**A**) In experiment 1, whole ovaries from twelve (*n* = 12) fish were allocated to one of four treatment groups. Ovaries cryopreserved using 1.3M dimethyl sulfoxide (DMSO) resulted in a higher cell viability in comparison to 1.3 M ethylene glycol (EG) and this was not significantly different to fresh control samples. The viability of the EG treated samples and the negative control samples were both significantly different compared to the fresh control (*p* = 0.018 and *p* = 0.0003 for Fresh vs EG, and Fresh vs Neg samples, respectively; minimum level of significance indicated by *). (**B**) In experiment 2, DMSO also was tested at concentrations of 1.0 M, 1.6 M and 2.0 M with nine ovaries (*n* = 9) allocated to one of the three treatments. No significant difference was found in cell viability between the different concentrations of DMSO and fresh controls (non-significance indicated by “ns”), but all samples were significantly different to the negative control (*p* < 0.0001, level of significance indicated by ****; One-way ANOVA with Tukeys post hoc test, data shown as mean ± standard deviation).

**Figure A1:**
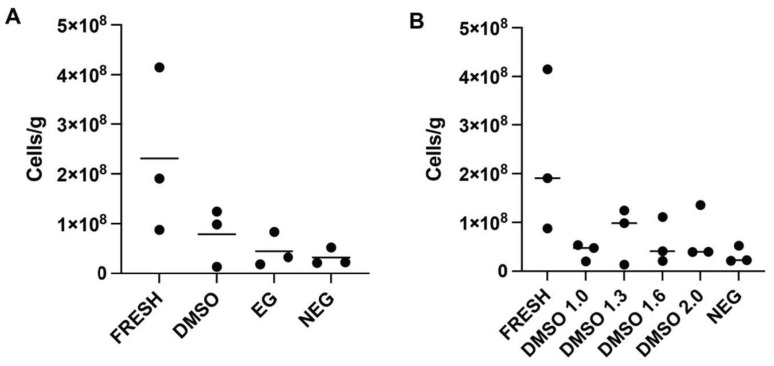
The number of cells recovered per gram of tissue was calculated for each treatment, (**A**) fresh tissue, tissue cryopreserved with DMSO and Ethylene Glycol (EG), and tissue cryopreserved in cryomedium with no permeating cryoprotectant (NEG), as well as (**B**) different concentrations of DMSO from 1.0 M to 2.0 M. No significant difference was reported in cells/g between treatment groups in graph “A” (*p* = 0.0905), and “B” (*p* = 0.0798; One-way ANOVA with Tukey’s post hoc test).

Comparison of cell viability in ovarian tissue cryopreserved in 1.3 M DMSO or 1.3 M EG showed that DMSO-treated samples had higher cell viability post-thaw at 63.5 ± 18.2% which was comparable to fresh samples (82.5 ± 7.1%; *p* = 0.36). EG treated samples has a significantly lower viability post-thaw (40.3 ± 17.9%) than fresh samples (*p* = 0.019), but were not significantly different to DMSO treated samples (*p* = 0.22). All treatments, DMSO, EG and fresh samples, were found to perform significantly better than the negative control (1.0 ± 0.5%; *p* < 0.027). As DMSO performed best in comparison to fresh controls, we tested cryomedia containing DMSO at 1.0 M, 1.6 M and 2.0 M compared to the original DMSO concentration of 1.3 M. No statistical significance was reported between the different concentrations compared to a fresh control. However, tissue cryopreserved in 2.0 M did have a higher cell viability (76.07 ± 3.89%) and a smaller range in viability across three replicates (Figure 4). The number of cells recovered per gram of tissue treated was assessed to ensure viability assessment was not skewed by samples in which dead cells had been entirely destroyed, this assessment revealed no significant differences in cell number recovered per gram of tissue between treatment groups (Figure A1). 

## 4. Discussion

We have described protocols for successful cryopreservation of ovarian tissue in the Murray River rainbowfish (*Melanotaenia fluviatilis*) and the isolation and viability analysis of ovarian cell suspensions using flow cytometry. Ovarian tissue from this species cryopreserved in the permeating cryoprotectant DMSO at concentrations between 1.0 to 2.0 M were all found to have viabilities comparable to fresh controls. This is consistent with our previous findings in testicular tissue in this species [22]. Furthermore, this is the first report of ovarian tissue cryopreservation within the order Atheriniformes and the family Melanotaeniidae, providing a baseline method to begin the investigation of similar use of this biobanking method in other species within these groups. 

Previous studies have used both slow cooling and vitrification methods to cryopreserve fish ovarian tissue. In transgenic rainbow trout, oogonia producing green fluorescence were isolated by flow cytometry and were found to have a viability of 72.9% when tissue was cryopreserved in 1.0 M DMSO, 0.1 M trehalose and 10% (*vol*/*vol*) egg yolk, using a slow cooling method [29]. In carp, slow cooling resulted in approximately 65% viability in oogonia post thaw, using a cryomedia containing 1.5 M DMSO [14]. Vitrification of ovarian tissue in salmonids [16] and the cyprinid honmoroko [19] resulted in oogonia viabilities of 37.98–40% and ~50%, respectively. While both slow cooling and vitrification have been used previously, slow cooling is preferable when working with threatened species as it is more flexible for in-field applications, requiring minimal equipment to begin the initial freezing process. 

Post-thaw viability assessment is usually performed using the trypan blue exclusion assay as seen in the aforementioned studies in rainbow trout, carp, salmonid and cyprinid honmoroko. Here, we used flow cytometry as a tool to isolate cell populations from our fresh and cryopreserved ovarian cell suspensions and analyse their viability. Flow cytometry uses a fluidics and laser system to individually interrogate each cell in a suspension, gathering information that can help identify unique cell populations and sort specific cell types out of suspension. The analysis and sorting of fish testicular cells using flow cytometry has been reported in numerous species including starry goby (*Asterropteryx semipunctata*) [30] Pacific blue fin tuna (*Thunnus orientalis*) [31], Japanese char (*Salvelinus leucomaenis*) and masu salmon (*Oncorhynchus masou*) [32]. We had also reported the use of flow cytometry to analyse testicular cell suspension in *M. fluviatilis* [22]. However, few studies have used this method to analyse ovarian cell suspensions in fish, with those that have reporting only in transgenic species [29]. Here, we have analysed fish ovarian cell suspensions in a non-transgenic, native species. To do this we first had to produce a sufficient number of single ovarian cells in suspension for analysis. Similar studies in male fish have produced cell suspensions by crushing testicular tissue with a glass tissue grinder [22,30]. Tissue grinders are a time-efficient method to produce cell suspensions, however initial assessment of this method in the *M. fluviatilis* ovary indicated that grinding alone was not sufficient to separate oogonia into suspension. Our histological analysis of the ovary of this species indicated that oogonia exist in closely bound nests within the germinal epithelium. To disrupt these tight bonds, we tested two digestion methods and found that ovarian cell suspensions produced using 0.25% trypsin with 1.13 mM EDTA in PBS yielded the highest overall recovery of cells per grams of tissue digested, and the highest cell viability. This has defined a successful method to produce a single cell suspension of ovarian cells in this species. These can be used in future downstream applications such as ovarian cell injection into a surrogate or in vitro cell culture. 

Flow cytometry is a fast and objective means of determining the viability of select population of cells. As oogonia were our primary focus in ovarian tissue cryopreservation, we wanted to determine if flow cytometry could be used to isolate oogonia from a single cell suspension. Other studies have often focused on gradient-based separation methods, with studies in smaller fish species such as zebrafish requiring ovaries from four or five individuals to produce a large enough cell volume to analyse [33]. Methods designed to isolate specific cell populations, such as flow cytometry, may be better suited to isolate oogonia from small amounts of gonadal tissue. This is particularly important when considering conservation applications where access to tissue is often very limited. In this study we found that unsorted samples of ovarian cells in suspension contained approximately 2.53 ± 1.31% oogonia, which was consistent with histological estimations of the total proportion of oogonia in the ovary of 2.29 ± 0.81%. After sorting ovarian cell suspensions using the flow cytometer, the number of oogonia increased to 5.85 ± 4.01%, almost doubling the number of oogonia available compared to unsorted controls. 

**Figure A2:**
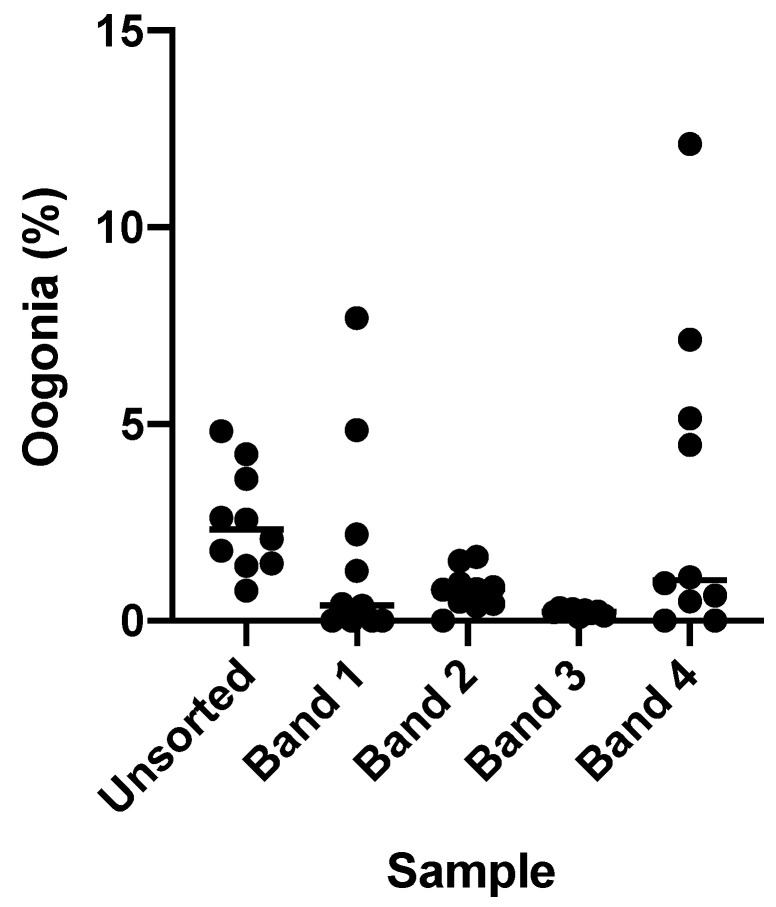
An ovarian tissue suspension (pooled, *n* = 2) separated on a 20%/40%/60% AllGrad gradient by centrifugation resulted in four cell bands (Bands 1–4). These cell bands were stained with anti-Vasa antibody and Hoechst and cells were counted across 10 fields. None of the cell bands resulted in a significantly higher proportion of oogonial cells in comparison to an unsorted control (Kruskal-Wallis with Dunn’s post-hoc test).

The advantage of gonad cryopreservation in comparison to gamete cryopreservation methods is that it can be performed using fish at any life stage, regardless of their sexual maturity and the natural spawning period of the fish. This is particularly important when considering in-field applications for cryopreservation methods, as techniques restricted to one phase of the reproductive cycle will significantly limit the number of animals that can be sampled. However, this can also result in variations in the number of oogonial cells available. The number of oogonia available in the ovary of our selected species was very low in comparison to previous studies, which have used immature or non-spawning individuals with a naturally higher proportion of oogonia present in the ovary. The fish used in this study were all of approximately reproductive age, with oocytes at various stages of oogenesis, and thereby contained a naturally low proportion of oogonial cells. It is therefore difficult to compare directly the recovery rate of this sorting method to previous studies. However, in our own pilot studies in this species, gradient centrifugation using a 20/40/60% AllGrad (CooperSurgical, Malov, Denmark) gradient did not result in any cell bands with an increased proportion of oogonia compared to an unsorted cell sample (Figure A2). Producing a sufficiently enriched sample of oogonia is important for the success of downstream applications such as germ cell transplantation, and this will be more challenging in species were total oogonia counts are low. Here, we have shown the usefulness and efficiency of flow cytometry to isolate and enhance the number of oogonia from ovarian cell suspensions in species with low numbers of oogonia in the ovary.

While the cryopreservation of fish ovarian tissue has been more successful in comparison to the cryopreservation of more differentiated germ cell types such as mature oocytes and even whole embryos, oogonia isolated by any method from the ovary must be able to differentiate into viable, fertilisable oocytes in order to be able to produce new individuals. Transplantation of fish ovarian cells into sterile surrogates using germ cell transplantation techniques has resulted in the production of oocytes in two species of fish; pejerrey (*Odontesthes bonariensis*) [34] and rainbow trout (*Oncorhynchus mykiss*) [35]. Interspecies transplantation of oogonia and primordial germ cells has shown that oogenesis appears to be sensitive to species-related differences between the donor oogonia and the surrogate ovarian environment, often resulting in transplant failure [4]. The production of oocytes relies on maternal depositing of germplasm and, later, vitellogenin to produce functional eggs [36]. A better understanding of the species-specific requirements of oogenesis will allow us to select better surrogate species for transplantation. The interspecies transplantation of oogonia maybe one method to identify the critical requirements needed for oogenesis to occur in a surrogate environment. Identifying the stage at which cell transplantation fails, will enable investigation of why this failure has occurred and help to determine ways in which to correct it or identify surrogates that may be more suitable. For example, if oogonia do not colonise the gonad at all, this may suggest important differences in cell signaling pathways between species. Alternatively, if oogonia are able to colonise the gonad but fail to enter the gonadotrophin-dependent secondary growth phase, endocrinological differences may be the cause of failure. In this way, the interspecies transplantation of oogonia is not just an assisted reproductive technique that may be useful in conservation or aquaculture, but a tool to study the critical processes of oogenesis in fish species. Surrogate selection and species relatedness will be particularly important in the context of conservation. Studies in frogs have shown closely related species often face similar environmental pressures and threats [37]. If the same is true of fish species, closely related surrogates may not always be available if these close relatives are also facing their own conservation concerns. A better understanding of the mechanisms behind oogenesis and species related conflicts that arise after germ cell transplantation, will inform better surrogate selection in future transplantation studies in endangered fish species.

## 5. Conclusions

In the absence of robust cryopreservation methods for fish oocytes and embryos, the cryopreservation of ovarian tissue from fish is the most accessible option for the biobanking of important maternal fish genetics using reproductive tissue. With the continued degradation of the freshwater environment, fish populations remain at risk of extinction. Here we have validated a protocol for the cryopreservation, isolation and handling of fish ovarian cells in *M. fluviatilis*. The family Melanotaeniidae contains a number of endangered and critically endangered relatives of the Murray River rainbowfish that could benefit from the use of biobanking as a means of securing these species against extinction. With fish species in decline, and limited opportunities to cryopreserve fish oocytes and embryos, the continued exploration of ovarian tissue cryopreservation and its use in reproductive techniques such as germ cell surrogacy is critical for the improvement of ex situ conservation methods in female fish. 

## Data Availability

The data presented in this study are available in the Supplementary Material.

## Supplementary Materials

The following supporting information can be downloaded at: https://www.mdpi.com/article/10.3390/ani12060794/s1, Data set: Cryopreservation of ovarian tissue in *Melanotaenia fluviatilis*.
